# General trust impedes perception of self-reported primary psychopathy in thin slices of social interaction

**DOI:** 10.1371/journal.pone.0196729

**Published:** 2018-05-02

**Authors:** Joseph H. Manson, Matthew M. Gervais, Gregory A. Bryant

**Affiliations:** 1 Department of Anthropology, University of California Los Angeles, Los Angeles, California, United States of America; 2 Center for Behavior, Evolution and Culture, University of California Los Angeles, Los Angeles, California, United States of America; 3 Department of Psychology, University of British Columbia, Vancouver, BC Canada; 4 Department of Communication, University of California Los Angeles, Los Angeles, California, United States of America; Universite de Bretagne Occidentale, FRANCE

## Abstract

Little is known about people’s ability to detect subclinical psychopathy from others’ quotidian social behavior, or about the correlates of variation in this ability. This study sought to address these questions using a thin slice personality judgment paradigm. We presented 108 undergraduate judges (70.4% female) with 1.5 minute video thin slices of zero-acquaintance triadic conversations among other undergraduates (targets: n = 105, 57.1% female). Judges completed self-report measures of general trust, caution, and empathy. Target individuals had completed the Levenson Self-Report Psychopathy (LSRP) scale. Judges viewed the videos in one of three conditions: complete audio, silent, or audio from which semantic content had been removed using low-pass filtering. Using a novel other-rating version of the LSRP, judges’ ratings of targets’ primary psychopathy levels were significantly positively associated with targets’ self-reports, but only in the complete audio condition. Judge general trust and target LSRP interacted, such that judges higher in general trust made less accurate judgments with respect to targets higher in primary and total psychopathy. Results are consistent with a scenario in which psychopathic traits are maintained in human populations by negative frequency dependent selection operating through the costs of detecting psychopathy in others.

## Introduction

Psychopathy is a syndrome characterized by pathological lying, manipulativeness, grandiosity, shallow emotions, impulsivity and irresponsibility, and lack of empathy and remorse [1, 2]. Psychopathy can be conceptualized as a trait continuum in the general population ([3, 4]; but see [5]). Among the “Dark Triad” traits [6], psychopathy is distinguished from Machiavellianism by a shorter-term temporal orientation, and from narcissism by a focus on instrumental goals rather than self-enhancement needs [7]. The oldest and most commonly used structural model of psychopathy ([8]; but see [9, 10]) posits two factors, typically correlated at 0.40–0.50. Primary psychopathy encompasses callous affect and a manipulative, deceitful interpersonal style, sometimes manifest as “glib charm.” Secondary psychopathy encompasses an erratic lifestyle, impulsivity, and antisocial behavior. In terms of the Big Five personality structure, both psychopathy factors are negatively related to agreeableness and conscientiousness, but only secondary psychopathy is also associated with low extraversion and high neuroticism [11]. Other divergent correlates of the two factors include (1) psychological distress (negatively associated with primary psychopathy and positively associated with secondary psychopathy) [12] and (2) a propensity toward instrumental aggression (i.e. goal-driven aggression motivated by attainment of an external reward), which is generally associated only with primary psychopathy; hostile/reactive aggression is positively associated with both primary and secondary psychopathy [13].

### Detecting psychopathy in others

How well can people detect the presence of psychopathic traits in others? In general, everyday judgments of others’ personality traits tend to be reasonably accurate [14], as would be expected given a long history of natural selection favoring accurate predictions of other people’s behavior [15]. However, accurate personality judgment is possible only when the target individual does something relevant to the trait being judged, in the presence of the judge, and this behavior is both detected and utilized correctly by the judge (The Realistic Accuracy Model [RAM]: [16]). Furthermore, judges’ own dispositions color their perceptions of others’ personalities, partly via the effects of assumed similarity bias [17, 18], and partly through more subtle causal pathways [19].

The ability to detect psychopathic traits based on limited information would have obvious adaptive value, enabling individuals to avoid entering relationships in which they would be exploited. However, the interpersonal style characteristic of primary psychopathy may be an adaptation that enables psychopathic individuals to escape detection, setting up a co-evolutionary process (“arms race”) between psychopathy detection and psychopathy concealment [20, 21]. Cleckley [1], writing primarily about clinical populations, noted that “…the typical psychopath will seem particularly agreeable and make a distinctively positive impression when he is first encountered. Alert and friendly in his attitude, he is easy to talk with…Nor does he, on the other hand, seem to be artificially exerting himself like one who is covering up or who wants to sell you a bill of goods.” Thus, primary psychopathy may differ from almost all other personality traits in that its detection is hindered, as a matter of evolved design, by aspects of the trait itself [22]. The capacity to detect psychopathy can be viewed as a specific form of the capacity to detect others’ propensities to defect in social exchanges, a topic of ongoing controversy [23–26].

In this paper, we examine the overall accuracy (operationalized as self-other agreement) of judgments of subclinical primary, secondary, and total psychopathy in undergraduate target individuals based on video thin slices of zero-acquaintance social interaction. A behavioral thin slice is a brief (< 5 minute) video or audio clip of a target individual engaged in social interaction or prompted monologue [27]. Compared to real-life acquaintances, judges of thin slices suffer from a dearth of valid and available personality cues, yet they do impressively well at achieving both inter-judge and judge-target agreement with respect to certain personality traits [28, 29]. Extraversion is the easiest of the Big Five dimensions for strangers to judge, followed by conscientiousness and agreeableness [29].

Research findings are mixed regarding whether psychopathic traits manifest themselves in behavioral thin slices. One study [30] has found that psychology student judges were significantly accurate at judging the self-reported and clinically-diagnosed psychopathy levels of male maximum-security inmates based on 5- and 10-second video excerpts of monologue. However, other research [22], using similar methods, found no significant accuracy in judgments of the psychopathy levels of incarcerated women, possibly because of sex differences in the extent to which the interpersonal style characteristic of primary psychopathy creates a likeable façade. Following a directed dyadic problem-solving task, undergraduate participants higher in self-reported psychopathy were perceived by their task partners as higher in dominance and low in nurturance, ingenuousness and conscientiousness [31]. However, participants in that study were not asked to assess each other specifically with regard to Dark Triad traits. Furthermore, some of the dyads were acquainted before the experiment.

### The present study

The present study is the first to examine directly whether the self-reported psychopathy levels of non-institutionalized target individuals can be detected based solely on behavioral thin slices. We predicted that judges would be more accurate assessing primary psychopathy than secondary psychopathy. This pattern is reported for judgments of clinical psychopathy levels of maximum-security inmates based on brief videotaped monologues [30]. Theoretically, primary psychopathic traits, compared to secondary psychopathic traits, are more likely to predispose individuals to exploit their social partners, thus placing a greater premium on the ability to detect them from first impressions. In terms of construct content, primary psychopathy is the psychopathy factor most closely linked to interpersonal style, suggesting that it will manifest itself more clearly than secondary psychopathy in the zero-acquaintance social interactions that comprise our stimuli. However, as discussed above, one proposed component of primary psychopathy is a convincing façade of sincere friendliness. This countervailing consideration suggests that primary psychopathy could be more difficult to detect than secondary psychopathy.

Building on the finding [30] that judgments of psychopathy were more accurate when judges viewed silent video than when they viewed video accompanied by audio, we included a silent video condition in our study design. Psychopathy levels may be correlated with purely visual cues, e.g. aspects of facial structure [32] and the creation of a physically attractive veneer [33]. We also included a video condition in which targets’ speech was low-pass filtered, which eliminates linguistic content by removing all frequencies above a specified cut-off point while retaining prosodic characteristics, including pitch, loudness, and speech rate dynamics. In the zero-acquaintance triadic conversations from which we drew our video stimulus materials (see Methods), self-reported primary psychopathy (but not secondary psychopathy) was positively correlated with the proportion of a triad’s words uttered [34]. The low-pass filtered video condition afforded the opportunity to test whether conversational dominance per se, irrespective of speech content, influenced judges’ assessments of targets’ psychopathy levels.

Individual differences among judges may affect judgments of targets’ psychopathy levels. Gillen et al. [22] found that judges higher in agreeableness and lower in the anti-social facet of psychopathy were more accurate in their thin slice-based judgments of primary psychopathy in incarcerated female targets, whereas judges higher in extraversion and lower in neuroticism and the disorganized lifestyle facet of psychopathy were more accurate in their judgments of secondary psychopathy. We examined whether three traits of judges were related to their judgments of targets’ psychopathy levels. General trust (an expectation that most people are motivated by good will and benign intent) may be distinguished from (lack of) caution, with caution defined as vigilance regarding exploiters, even if one believes that they are rare [35]. We predicted that judges who were both high in general trust and low in caution would rate targets as lower in psychopathic traits, compared to other judges. As described in Results, our caution instrument failed to show adequate internal reliability. We therefore tested whether general trust alone predicted (1) psychopathy judgments and (2) discrepancies between judged psychopathy levels and targets’ self-reported psychopathy levels.

We also tested a prediction which, although consistent with our general approach, was not part of our original study design but rather was suggested by a reviewer of an earlier version of this paper. Adaptive error management theory [36] suggests that people will generally overestimate the potential dangerousness of strangers (reviewed in [22]) because the costs of false alarms are lower than the costs of mistaken attributions of harmlessness. Individual differences in trade-offs between the costs of false alarms and misses (as defined in signal detection theory [37]) in this domain may be reflected in individual differences in general trust. In other words, judges low in general trust might overestimate the psychopathy levels of most target individuals, but make more accurate judgments than high-trusters with respect to targets who are high in psychopathic traits.

Finally, because our thin slice videos depicted social interaction, and because empathy is generally associated with greater interpersonal sensitivity [38], we reasoned that more empathetic judges would make more accurate judgments of targets’ psychopathy levels. Thus, we tested whether the trait empathy level of judges was related to the accuracy of their judgments of targets’ psychopathy levels. Empathy comprises two components: affective empathy (appropriate emotional responses to another’s affective state) and cognitive empathy (understanding another’s affective or other internal state) [39]. We made no prediction regarding which component would be more closely related to judgment accuracy. We also made no predictions about differences between experimental conditions in the relationships between judge general trust or empathy and accuracy at detecting psychopathy.

## Materials and methods

### Judges and judge self-reports

Judges (n = 108, 70.4% female, ages and ethnicities unknown) were University of California, Los Angeles, undergraduates fulfilling a course research participation requirement. Judges were recruited five years after the last target individuals (see below) completed participation. Judges completed exactly three self-report instruments. The 6-item General Trust Scale (exemplary item: “Most people are basically honest”) and 7-item Caution Scale (exemplary item: “There are many hypocrites in this society”) elicit responses on a 5-point scale [35]. We measured social perceptiveness and empathy using the 26-item version of the Empathy Quotient (EQ) [40]. Responses are made on a 4-point scale. Items include “I am quick to spot when someone in a group is feeling awkward or uncomfortable.” Eight items tap cognitive empathy (the ability to attribute mental states to others), 8 items tap affective empathy (appropriate emotional responses to others’ mental states), 4 items tap social skills, and 6 items do not belong to any subscale. All procedures involving the judges were approved by the UCLA Institutional Review Board (Approval #15–000238), and written informed consent was obtained from all participants.

### Thin slice stimuli and target self-reports

Video stimuli consisted of the first 1.5 minutes of each of 35 10-minute 3-person same-sex zero-acquaintance conversations among UCLA students. We chose a 1.5 minute slice duration because it adequately samples the frequencies of a wide range of behaviors [41]. We viewed the videos and confirmed that every target individual spoke at least once (beyond merely introducing themselves) during the first 1.5 minutes of their interaction. Target individuals (n = 105, 57.1% female, mean age = 19.6, SD = 3.6) were informed of the videotaping and were told that the study topic was “Small Talk Among Strangers” and that they were free to discuss whatever they wished. The target sample was 35.2% Asian or Asian-American, 34.3% White, 8.6% mixed White-Asian, 4.8% African-American, 4.8% Latino/a, 4.8% mixed White-Latino, 2.9% Pacific Islander, 1.9% Middle Eastern, 1.0% mixed White-Pacific Islander, 1.0% mixed Asian-Pacific Islander, 1.0% self-identified as “Other.” Among other tasks following the conversation, targets completed the Levenson Self-Report Psychopathy Scale [42], a 26-item instrument that produces the expected two-factor structure and is both reliable and externally valid [43]. Responses are made on a 4-point scale. The 16 items tapping primary psychopathy include “I enjoy manipulating other people’s feelings.” The 10 items tapping secondary psychopathy include “I don’t plan anything very far in advance.” The LSRP was the only self-report personality instrument completed by the target individuals. For additional details regarding the target individuals and the procedure in which they participated, see [44]. All procedures involving the target individuals were approved by the UCLA IRB (Approvals #G07-10-097-01 to -04), and written informed consent was obtained from all participants.

### Low-pass filtered stimuli

Uncompressed audio files were extracted from videos and low-pass filtered at 0.5 kHz using a Butterworth filter (24 dB/octave roll off, 100 Hz transition bandwidth) in Adobe Audition 3.0. Following the filtering process, files were amplitude normalized. This level of filtering reduces lexical identification to near-zero while retaining energy at the fundamental frequency (i.e., the acoustic correlate of perceived pitch), the first formant (i.e., speech frequency band that contributes to phonetic judgments), as well as amplitude and speech rhythm dynamics [45, 46].

### Procedure

All judges first completed the General Trust and Caution scales and the EQ. They were then shown the 26 LSRP items, grammatically altered into other-report items, e.g. “She enjoys manipulating other people’s feelings.” We will refer to this scale as the LORP (Levenson Other-Report Psychopathy scale). Each judge then viewed three 1.5 minute videos, all of them in the same stimulus condition (video with full audio, silent video, or low-pass filtered video). Each judge viewed nine target individuals of one sex (not necessarily the same sex as the judge). Judges were informed only that the target individuals were meeting for the first time; they were not told about the experimental procedures that followed the videotaped interaction (described in [44]]. Judges completed the LORP after each video with respect to each of its three target individuals. Each target was judged by nine judges (three in each stimulus condition). Judges were instructed to inform the experimenter if they recognized any of the target individuals (this never happened). The order in which triads were presented was counterbalanced, and the order of LORP completion was counterbalanced among target individuals within each triad of targets. Videos were assigned to judges such that, within each condition, no pair of triads was viewed together by more than one judge.

### Data analysis

Targets’ LSRP scores, judges’ General Trust, Caution and Empathy Quotient scores, and judges’ rating of targets (i.e. LORP scores) were calculated as the means among all non-missing items of the relevant scale. The data set consisted of 945 judge-target pairings (315 in each stimulus condition) for each of the two psychopathy factors and for total psychopathy (i.e. mean scores on all 26 LSRP/LORP items). Statistical analyses were carried out using Stata 14.1. We tested predictions using multilevel mixed effects linear regression models. These models represent a generalization of linear regression that allows for both fixed (hypothesis-driven) effects and random effects, such as participant identity, other than those associated with the overall error term. Thus, multiple observations from the same individual may be treated as data points without assuming their independence from each other. As originally planned, the random factors in our models were judge ID, target ID and conversation triad ID. Target ID was nested within triad ID, and triad ID was crossed with judge ID. However, the variance of the triad effects was consistently very close to zero, and removing triad as a random factor had almost no effect on the parameter estimates of the fixed effects. We therefore report the results of models in which target ID and judge ID (crossed) are the only random effects. All continuous variables were standardized (converted to z scores) before analysis. Thus, our reported coefficients are the equivalents of standardized regression coefficients. In the first set of planned analyses, to assess the influence of psychopathic traits on judgments, the dependent variable was the judge’s LORP score, and the fixed factors included the target’s self-reported (i.e. LSRP) score and the judge’s General Trust score. In the second set of planned analyses, to assess correlates of judgment accuracy, the dependent variable was the absolute value of the difference between the judge’s LORP score and the target’s LSRP score, where positive coefficients indicate greater deviation from the self-reported score, i.e. lower accuracy. The fixed factors were judge EQ and judge General Trust. In the third set of analyses, to examine whether higher General Trust in judges is associated with lower levels of perceived target psychopathy relative to self-reported psychopathy, our dependent measure was the standard difference score (SDS) [47] between LSRP and LORP score. SDS is calculated by subtracting the z score of one rating of a target (here, the self-report) from the z-score of another rating of that target (here, the judge’s rating). Thus, a positive SDS score indicates that a judge has rated a target’s relative psychopathy level higher than the target’s self-report, whereas a negative SDS score indicates the reverse. In all three sets of analyses, we used the Akaike Information Criterion (AIC) and Bayes Information Criterion (BIC) to compare all models predicting the same dependent variable in the same experimental condition. In a comparison between two models predicting the same dependent variable, lower AIC and BIC scores indicate a better approximation to the underlying causal processes without over-fitting to the data. BIC, compared to AIC, penalizes models more for additional parameters. For effects that attained conventional levels of statistical significance (p < 0.05) in multivariate models, effect size (Cohen’s f^2^) was calculated using a technique published for use with SAS [48], as adapted for Stata by Andy Lin (personal communication) of the UCLA Institute for Digital Research and Education.

Because target ethnicity was unexpectedly found to be related to psychopathy judgments (see Results), we report these results in addition to those that bear on our hypotheses.

The data analyzed for this study, and an example of the Stata Do-Files used to run the multilevel mixed effects regression models, can be accessed in the Dryad repository at https://doi.org/10.5061/dryad.b49n840.

## Results

### Descriptive statistics and reliability

Table 1 shows descriptive statistics for all variables, including target self-report and judge self-report measures, and judges’ ratings of targets. Although our analyses used averaged item scores from the LSRP scales, we also report summed item scores to permit comparison of our results to previous research that used summed LSRP item scores [42, 49]. Responses were missing for 0.7% of LSRP responses, 0.1% of LORP responses, 0.2% of General Trust responses, 0.1% of Caution responses, and 0.1% of EQ responses. LSRP scores were lower than targets’ average (across all raters) LORP scores (n = 105; primary psychopathy, t = 9.53, p < 0.0001, d = 1.27; secondary psychopathy, t = 10.69, p < 0.0001, d = 1.34; total psychopathy, t = 11.62, p < 0.0001, d = 1.51). Table 2 shows scale reliabilities of the three judge self-report measures, and correlations among them. Because the Caution scale showed unacceptably low reliability, we excluded Caution from our model tests. LORP scores showed high inter-item reliability for both primary (α = 0.89) and secondary psychopathy (α = 0.78). The correlation between LSRP F1 and F2 scores was 0.38 (p < 0.001) [44]. Correlations between LORP F1 and F2 scores are shown in Table 3.

**Table 1:** Descriptive statistics for target and judge self-report measures and ratings.

Measure	Mean (SD)	n	Skweness	Kurtosis
**Target self-report**				
LSRP^a^ F1^b^ (sum)	30.31 (7.47)	98	0.79	3.80
LSRP^a^ F1^b^ (mean)	1.90 (0.48)	105	0.69	3.55
LSRP^a^ F2^c^ (sum)	18.59 (3.99)	98	0.27	3.29
LSRP^a^ F2^c^ (mean)	1.87 (0.41)	105	0.19	3.09
Total LSRP^a^ (sum)	48.96 (9.63)	97	0.83	3.37
Total LSRP^a^ (mean)	1.89 (0.38)	105	0.71	3.19
**Judge self-report**				
General trust	3.44 (0.50)	108	-0.52	3.01
Caution	3.15 (0.41)	108	0.25	3.38
Empathy quotient	2.94 (0.26)	108	0.08	3.38
**Judge ratings of targets: all conditions**				
LORP^d^ F1^b^	2.36 (0.42)	945	0.15	3.51
LORP^d^ F2^c^	2.29 (0.40)	945	-0.00	3.53
Total LORP^d^	2.34 (0.38)	945	-0.01	3.59
**Judge ratings of targets: video with full audio**				
LORP^d^ F1^b^	2.32 (0.38)	315	0.18	3.10
LORP^d^ F2^c^	2.25 (0.38)	315	0.37	3.24
Total LORP^d^	2.29 (0.34)	315	0.16	3.37
**Judge ratings of targets: silent video**				
LORP^d^ F1^b^	2.37 (0.44)	315	0.17	3.95
LORP^d^ F2^c^	2.33 (0.42)	315	-0.24	4.08
Total LORP^d^	2.35 (0.40)	315	-0.10	4.14
**Judge ratings of targets: low-pass filtered video**				
LORP^d^ F1^b^	2.39 (0.42)	315	0.07	3.18
LORP^d^ F2^c^	2.31 (0.39)	315	-0.11	3.28
Total LORP^d^	2.36 (0.38)	315	-0.09	2.99

^a^Levenson Self-Report Psychopathy scale.

^b^Primary psychopathy.

^c^Secondary psychopathy.

^d^Levenson Other-Report Psychopathy scale.

**Table 2:** Scale reliability and bivariate correlations among judge self-report measures.

Measure	1.	2.	3.
1. General trust	**.69**		
2. Caution	-.20*	**.43**	
3. Empathy quotient	.07	-.07	**.75**

Cronbach’s alphas are bolded.

* p < 0.05

**Table 3:** Correlations between LORP F1 and F2 scores.

Condition	r	n	p
Video with full audio	0.57	315	<0.0001
Silent video	0.71	315	<0.0001
Video with low-pass filtered audio	0.70	315	<0.0001

### Target age and ethnicity and other-report psychopathy scores

Target age was unrelated to LORP scores in all conditions. However, target ethnicity was related to LORP scores. The multiplicity of target ethnicities yielded a large number of ways to treat this pattern analytically. As a dichotomous variable, ethnicity had its strongest relationship to LORP when it was coded as White or part White (i.e. White plus White-Asian plus White-Latino plus White-Pacific Islander, n = 51) vs. Non-White (all others, n = 54). For example, with respect to total psychopathy, and treating each judgment as independent without taking account of the clustering (random) variables, White target individuals were judged to be more psychopathic than Non-Whites in all three conditions: video with full audio (n_white_ = 153, n_non-white_ = 162, M_white_ = 0.01, SD = 0.90, M_non-white_ = -0.25, SD = 0.88, t = -2.62, p = 0.009, d = -0.30); silent video (M_white_ = 0.19, SD = 0.97, M_non-white_ = -0.12, SD = 1.12, t = -2.57, p = 0.011, d *=* -0.29); and low-pass filtered video (M_white_ = 0.33, SD = 1.04, M_non-white_ = -0.20, SD = 0.89, t = -4.91, p < 0.0001, d *=* -0.55). We therefore incorporated this dichotomous ethnicity variable into our model comparisons where LORP scores were the dependent variable. Ethnicity had a significant effect in only one set of models of judgment accuracy (absolute value of LORP-LSRP difference for total psychopathy). Therefore, we do not include ethnicity in the tables showing the results of the other judgement accuracy models. Also, because ethnicity was a covariate rather than a hypothesis-based independent variable, we do not report the results of models in which ethnicity was the sole predictor of LORP scores. In contrast to video-based judgments (LORP), self-reported psychopathy (LSRP) did not differ significantly between Whites and non-Whites (primary psychopathy, n_white_ = 51, n_non-white_ = 54, M_white_ = 0.10, SD = 0.97, M_non-white_ = -0.08, SD = 1.03, t = -0.92, p = 0.36, d = -0.18; secondary psychopathy, M_white_ = 0.07, SD = 1.14, M_non-white_ = -0.09, SD = 0.85, t = -0.80, p = 0.43, d = -0.15; total psychopathy, M_white_ = 0.10, SD = 1.05, M_non-white_ = -0.10, SD = 0.95, t = -1.04, p = 0.30, d = -0.20).

### Multi-level mixed regression models predicting other-report psychopathy scores

Standardized residuals of all models followed a standard normal distribution, indicating good model fit. Table 4 shows the results of tests of models predicting judges’ LORP ratings of targets on primary (F1) psychopathy. In the video with full audio condition, the best fitting model, as evaluated by AIC, included all three predictors (target F1 LSRP, target ethnicity, and judge general trust). However, only target ethnicity had a significant effect, such that White targets were judged to be higher in primary psychopathy than Non-White targets. The best fitting model, as evaluated by BIC, included only target LSRP: target individuals who described themselves as higher in primary psychopathy were described by judges as being higher in primary psychopathy. In the silent video condition, judges higher in General Trust rated targets as lower in primary psychopathy, whereas target LSRP and ethnicity were unrelated to LORP score. Neither of the hypothesized independent variables was related to LORP scores in the low-pass filtered video condition, but White targets were judged to be significantly higher in primary psychopathy than Non-White targets.

**Table 4:** Multilevel mixed regression models predicting Levenson other-report primary psychopathy judgments (LORP F1).

	Fixed factors						Random factors	
Condition	Target LSRP 1	Target ethnicity	Judge trust	Model χ^2^	ΔBIC^a^	ΔAIC^b^	Judge ID	Target ID
**Video with Full Audio**								
	0.09±0.05	0.22±0.10* (0.02)	-0.17±0.09	13.5**	2.8	0	0.23±0.07	0.08±0.04
	0.09±0.05	0.23±0.10* (0.02)		9.65**	0.6	1.6	0.26±0.07	0.08±0.04
	0.10±0.05* (0.01)		-0.17±0.09* (<0.01)	8.13*	2.1	3.0	0.22±0.07	0.09±0.03
		0.24±0.10* (0.02)	-0.17±0.09	9.64**	0.5	1.4	0.23±0.07	0.09±0.04
	0.10±0.05*			4.15*	0	4.7	0.25±0.07	0.09±0.04
			-0.18±0.09*	3.92*	0.3	5.0	0.23±0.07	0.11±0.04
**Silent Video**								
	-0.02±0.06	0.24±0.13	-0.26±0.09** (0.02)	12.89**	8.0	1.9	0.14±0.06	0.15±0.06
	-0.04±0.06	0.25±0.13		3.96	10.4	8.0	0.21±0.07	0.14±0.06
	-0.01±0.06		-0.26±0.08** (0.02)	9.71**	5.7	3.3	0.14±0.06	0.17±0.06
		0.23±0.13	-0.26±0.09** (0.02)	12.82**	2.4	0	0.14±0.06	0.15±0.06
	-0.02±0.06			0.15	8.4	9.7	0.20±0.07	0.16±0.06
			-0.26±0.08**	9.71**	0	1.3	0.13±0.06	0.17±0.06
**Low-pass Filtered Video**								
	0.01±0.07	0.51±0.11*** (0.07)	-0.00±0.06	21.14***	5.8	2.0	0.06±0.04	0.03±0.05
	0.02±0.07	0.51±0.11*** (0.07)		21.10***	0.04	0.04	0.06±0.04	0.03±0.05
	0.02±0.06		-0.00±0.07	0.13	19.2	19.2	0.07±0.05	0.09±0.06
		0.51±0.11*** (0.07)	0.01±0.07	21.14***	0	0	0.06±0.04	0.03±0.05
	0.02±0.06			0.12	13.4	17.2	0.07±0.05	0.09±0.06
			-0.00±0.07	0.00	13.6	17.3	0.07±0.05	0.09±0.06

Cells under Fixed factors show parameter estimates ± SE. Values in parentheses are estimated effect sizes (Cohen’s f^2^). For target ethnicity, Non-White = 0, White = 1. Cells under Random factors show estimated variance ± SE.

* p < .05.

** p < .01.

*** p < .001.

^a^Bayes Information Criterion difference from best model.

^b^Aikaike Information Criterion difference from best model.

The finding, in some models, that judgments were significantly positively influenced by target primary psychopathy only in the video with full audio condition raises the possibility that judges based their ratings on a common form of verbal content: target individuals’ revelations of their academic major. Several of the LSRP F1 items pertain directly to material acquisitiveness, so it might be that judges simply gave higher LORP F1 scores to targets who indicated that they were majoring in business or economics. However, targets who announced these majors were not judged to be significantly higher in primary psychopathy than other targets (n_business_ = 13, n_other_ = 86, M_business_ = 0.04, SD = 0.67, M_other_ = -0.12, SD = 0.59, t = -0.85, p = 0.40, d = 0.25). Another possible explanation for the finding of a significant LSRP-LORP association in the video with full audio condition is that it was driven entirely by sex differences in both self-reported and other-perceived primary psychopathy. However, although LSRP (self-report) F1 scores were higher in men than in women (n_women_ = 60, n_men_ = 45, M_women_ = -0.20, SD = 0.80, M_men_ = 0.28, SD = 1.17, t = 2.45, p = 0.016, d = 0.48), LORP F1 scores did not differ as a function of target sex in the video with full audio condition; indeed, there was a non-significant trend for judges to rate women as higher in primary psychopathy than men (n_women_ = 60, n_men_ = 45, M_women_ = -0.09, *SD* = 1.18, M_men_ = -0.16, SD = 0.85, t = -0.34, p = 0.37, d = 0.07).

Table 5 shows the results of tests of models predicting judges’ LORP ratings of targets on secondary (F2) psychopathy. In the video with full audio condition, judge General Trust was related to LORP score, such that more trusting judges rated targets lower in secondary psychopathy. White targets were judged higher in secondary psychopathy than Non-White targets. In the silent video condition, only target ethnicity was related to LORP F2 score. In the low-pass filtered video condition, White targets were judged higher in secondary psychopathy than Non-White targets, and target self-report was related (positively) to LORP score.

**Table 5:** Multilevel mixed regression models predicting Levenson other-report secondary psychopathy judgments (LORP F2).

	Fixed factors						Random factors	
Condition	Target LSRP 1	Target ethnicity	Judge trust	Model χ^2^	ΔBIC^a^	ΔAIC^b^	Judge ID	Target ID
**Video with Full Audio**								
	0.04±0.05	0.26±0.11* (0.02)	-0.20±0.06** (0.02)	16.86***	5.1	1.3	0.07±0.04	0.06±0.05
	0.05±0.05	0.27±0.11* (0.02)		7.12*	7.8	7.8	0.11±0.05	0.07±0.05
	0.06±0.06		-0.21±0.06** (0.02)	11.04*	10.7	7.0	0.06±0.04	0.07±0.05
		0.27±0.11* (0.02)	-0.20±0.06** (0.02)	16.02***	0	0	0.07±0.04	0.06±0.05
	0.06±0.06			1.00	7.9	11.7	0.10±0.05	0.09±0.05
			-0.20±0.06**	9.92**	0.2	3.9	0.06±0.04	0.08±0.05
**Silent Video**								
	0.10±0.06	0.41±0.12*** (0.04)	-0.09±0.08	16.03**	4.5	0.7	0.08±0.05	0.08±0.06
	0.10±0.06	0.41±0.12*** (0.04)		14.73***	0	0	0.09±0.05	0.08±0.06
	0.11±0.06		-0.09±0.08	4.52	9.4	9.4	0.08±0.05	0.12±0.07
		0.43±0.12*** (0.04)	-0.09±0.08	13.24**	1.2	1.2	0.08±0.05	0.09±0.06
	0.11±0.06			3.18	4.9	8.7	0.09±0.05	0.12±0.07
			-0.09±0.08	1.44	6.6	10.4	0.08±0.05	0.14±0.07
**Low-pass Filtered Video**								
	0.12±0.06* (0.01)	0.47±0.11*** (0.05)	0.07±0.07	23.60***	4.9	1.2	0.08±0.04	0.07±0.05
	0.12±0.06* (0.02)	0.47±0.11*** (0.05)		22.90***	0	0	0.09±0.04	0.07±0.05
	0.14±0.06* (0.02)		0.06±0.08	5.61	15.3	15.3	0.09±0.05	0.12±0.06
		0.49±0.12*** (0.05)	0.07±0.07	18.60***	3.4	3.4	0.08±0.04	0.08±0.05
	0.14±0.06*			5.11*	10.1	13.8	0.09±0.05	0.12±0.06
			0.06±0.07	0.59	14.5	18.2	0.09±0.05	0.14±0.06

Cells under Fixed factors show parameter estimates ± SE. Values in parentheses are estimated effect sizes (Cohen’s f^2^). For target ethnicity, Non-White = 0, White = 1. Cells under Random factors show estimated variance ± SE.

* p < .05.

** p < .01.

*** p < .001.

^a^Bayes Information Criterion difference from best model.

^b^Aikaike Information Criterion difference from best model.

Table 6 shows analogous model comparisons for total psychopathy. Judge general trust and target ethnicity were related to LORP score in the video with full audio and the silent video conditions, whereas only target ethnicity was related to LORP score in the low pass filtered video condition.

**Table 6:** Multilevel mixed regression models predicting Levenson other-report total psychopathy judgments.

	Fixed factors						Random factors	
Condition	Target LSRP total	Target ethnicity	Judge trust	Model χ^2^	ΔBIC^a^	ΔAIC^b^	Judge ID	Target ID
**Video with Full Audio**								
	0.06±0.05	0.26±0.10** (0.02)	-0.20±0.08* (0.01)	15.85**	4.2	0.5	0.17±0.05	0.07±0.04
	0.06±0.05	0.27±0.10** (0.02)		9.47**	4.2	4.2	0.21±0.06	0.08±0.04
	0.08±0.05		-0.20±0.08* (0.01)	8.78*	5.2	5.2	0.16±0.05	0.09±0.05
		0.27±0.10** (0.02)	-0.20±0.08** (0.01)	14.02***	0	0	0.17±0.06	0.08±0.04
	0.08±0.05			2.15	5.4	9.1	0.20±0.06	0.09±0.07
			-0.20±0.08*	6.45*	1.6	5.3	0.17±0.05	0.10±0.04
**Silent Video**								
	0.01±0.06	0.33±0.13** (0.02)	-0.21±0.08** (0.01)	13.38**	5.7	2.0	0.13±0.06	0.14±0.06
	0.00±0.06	0.34±0.13** (0.02)		6.98*	6.0	6.0	0.17±0.07	0.14±0.06
	0.03±0.07		-0.22±0.08** (0.02)	7.02*	6.4	6.4	0.12±0.05	0.17±0.07
		0.33±0.13** (0.02)	-0.21±0.08** (0.01)	13.35**	0	0	0.13±0.06	0.14±0.06
	0.02±0.07			0.09	6.9	10.7	0.16±0.06	0.16±0.07
			-0.22±0.08**	6.81**	0.8	4.5	0.12±0.05	0.17±0.07
**Low-pass Filtered Video**								
	0.05±0.06	0.51±0.11*** (0.07)	0.04±0.07	22.79***	5.5	1.8	0.08±0.05	0.04±0.05
	0.05±0.06	0.51±0.11*** (0.06)		22.58***	0	0	0.08±0.05	0.04±0.05
	0.07±0.06		0.02±0.08	1.56	18.8	18.8	0.09±0.05	0.11±0.06
		0.52±0.11*** (0.07)	0.03±0.07	21.76***	0.6	0.6	0.08±0.05	0.04±0.05
	0.07±0.06			1.48	13.1	16.9	0.09±0.05	0.10±0.06
			0.02±0.08	0.07	14.5	18.3	0.09±0.05	0.11±0.06

Cells under Fixed factors show parameter estimates ± SE. Values in parentheses are estimated effect sizes (Cohen’s f^2^). For target ethnicity, Non-White = 0, White = 1. Cells under Random factors show estimated variance ± SE.

* p < .05.

** p < .01.

*** p < .001.

^a^Bayes Information Criterion difference from best model.

^b^Aikaike Information Criterion difference from best model.

### Models predicting accuracy (absolute value of self-other differences) in psychopathy judgments

Tables 7–9 depict models, from the original data analysis plan, of judge characteristics predicted to affect target psychopathy judgment accuracy, operationalized as absolute difference between LORP and LSRP score. In no condition did the Empathy Quotient have the predicted positive effect on accuracy, and in the silent video condition, judges higher in empathy actually made significantly less accurate judgments. Separate analyses of the cognitive and affective empathy subscales of the EQ also failed to find any positive effects of empathy on judgment accuracy. In the silent video condition, substituting into the models one EQ subscale (cognitive empathy or affective empathy) for the full EQ revealed that the negative effect of empathy on judgment accuracy was attributable more to cognitive empathy than to emotional empathy. For judgments of primary psychopathy, the parameter estimate for cognitive empathy was 0.14 ± 0.05 (p = 0.007), whereas for affective empathy it was 0.06 ± 0.05 (p = 0.25). Corresponding parameter estimates for judgments of secondary psychopathy were 0.11 ± 0.05 (p = 0.03) and 0.09 ± 0.05 (p = 0.07). In the video with full audio condition, judges higher in General Trust made less accurate judgments of primary psychopathy. This result is not due to a ceiling effect (more trusting judges’ ratings concentrated at the low end of the LORP scale), because even the most trusting quartile of full audio condition judges (n = 9) rated target individuals (mean of means) at 2.28 (range: 1.75–2.81) on the 4-point LORP primary psychopathy scale. Table 8 shows analogous analyses for secondary psychopathy. As we found for judgments of primary psychopathy, greater empathy predicted less accurate judgments only in the silent video condition. No other effects of judge characteristics on accuracy were found. With respect to total psychopathy (Table 9), target ethnicity had a significant effect in the low pass filtered video condition, such that judgments of White targets’ psychopathy levels were less accurate than judgments of Non-White targets’ psychopathy levels.

**Table 7:** Multilevel mixed regression models predicting absolute value of LORP-LSRP difference for primary psychopathy.

	Fixed factors					Random factors	
Condition	Judge trust	Judge EQ	Model χ^2^	ΔBIC^a^	ΔAIC^b^	Judge ID	Target ID
**Video with Full Audio**							
	0.10±0.05	0.03±0.06	4.37	5.5	1.7	0.04±0.02	0.13±0.04
	0.10±0.05*		4.09*	0	0	0.04±0.02	0.13±0.04
		0.05±0.06	0.65	3.2	5.2	0.05±0.02	0.13±0.04
**Silent Video**							
	0.02±0.05	0.10±0.04* (0.02)	5.39	5.5	1.8	0.02±0.02	0.21±0.06
	0.02±0.06		0.16	4.6	4.6	0.04±0.03	0.22±0.06
		0.10±0.04*	5.16*	0	0	0.02±0.03	0.22±0.10
**Low-pass Filtered Video**							
	-0.02±0.05	0.08±0.05	2.38	N.A.^c^	N.A.^c^	0.01±0.03	0.20±0.06
	-0.00±0.05		0.00	N.A.^c^	N.A.^c^	0.19±0.05	0.02±0.03
		0.07±0.05	2.18	N.A.^c^	N.A.^c^	0.20±0.05	0.01±0.03

Cells under Fixed factors show parameter estimates ± SE. Values in parentheses are estimated effect sizes (Cohen’s f^2^). Cells under Random factors show estimated variance ± SE.

* p < 0.05.

^a^Bayes Information Criterion difference from best model.

^b^Aikaike Information Criterion difference from best model.

^c^Not applicable: no model significantly predicts outcome variable.

**Table 8:** Multilevel mixed regression models predicting absolute value of LORP-LSRP difference for secondary psychopathy.

	Fixed factors					Random factors	
Condition	Judge trust	Judge EQ	Model χ^2^	ΔBIC^a^	ΔAIC^b^	Judge ID	Target ID
**Video with Full Audio**							
	-0.06±0.05	0.04±0.06	1.97	N.A.^c^	N.A.^c^	0.02±0.02	0.18±0.05
	-0.05±0.05		1.33	N.A.^c^	N.A.^c^	0.02±0.02	0.18±0.05
		0.03±0.06	0.33	N.A.^c^	N.A.^c^	0.02±0.02	0.19±0.05
**Silent Video**							
	-0.01±0.05	0.09±0.04* (0.02)	4.66	5.7	1.9	0.02±0.02	0.20±0.05
	-0.02±0.06		0.08	4.1	4.1	0.04±0.03	0.20±0.05
		0.09±0.04*	4.59*	0	0	0.02±0.02	0.20±0.05
**Low-pass Filtered Video**							
	0.05±0.05	0.01±0.05	1.15	N.A.^c^	N.A.^c^	0.02±0.02	0.12±0.04
	0.05±0.05		1.13	N.A.^c^	N.A.^c^	0.02±0.02	0.13±0.04
		0.02±0.05	0.16	N.A.^c^	N.A.^c^	0.20±0.02	0.13±0.04

Cells under Fixed factors show parameter estimates ± SE. Values in parentheses are estimated effect sizes (Cohen’s f^2^). Cells under Random factors show estimated variance ± SE.

* p < 0.05.

^a^Bayes Information Criterion difference from best model.

^b^Aikaike Information Criterion difference from best model.

^c^Not applicable: no model significantly predicts outcome variable.

**Table 9:** Multilevel mixed regression models predicting absolute value of LORP-LSRP difference for total psychopathy.

	Fixed factors						Random factors	
Condition	Judge trust	Target ethnicity	Judge EQ	Model χ^2^	ΔBIC^a^	ΔAIC^b^	Judge ID	Target ID
**Video with Full Audio**								
	0.06±0.04	0.01±0.11	-0.00±0.05	2.22	N.A.^c^	N.A.^c^	0.00±0.02	0.17±0.05
	0.06±0.04	0.01±0.11		2.22	N.A.^c^	N.A.^c^	0.00±0.02	0.17±0.05
	0.06±0.04		-0.00±0.05	2.21	N.A.^c^	N.A.^c^	0.00±0.02	0.17±0.05
		-0.01±0.11	0.01±0.05	0.06	N.A.^c^	N.A.^c^	0.01±0.02	0.17±0.05
	0.06±0.04			2.21	N.A.^c^	N.A.^c^	0.00±0.02	0.17±0.05
			0.01±0.05	0.05	N.A.^c^	N.A.^c^	0.01±0.02	0.17±0.05
**Silent Video**								
	0.02±0.06	0.01±0.11	0.09±0.05	3.76	N.A.^c^	N.A.^c^	0.05±0.03	0.14±0.05
	0.02±0.07	0.02±0.11		0.14	N.A.^c^	N.A.^c^	0.06±0.03	0.14±0.05
	0.02±0.06		0.09±0.05	3.76	N.A.^c^	N.A.^c^	0.05±0.03	0.14±0.05
		0.01±0.11	0.09±0.05	3.60	N.A.^c^	N.A.^c^	0.05±0.03	0.14±0.05
	0.02±0.07			0.10	N.A.^c^	N.A.^c^	0.05±0.03	0.14±0.05
			0.09±0.05	3.61	N.A.^c^	N.A.^c^	0.05±0.03	0.14±0.05
**Low-pass Filtered Video**								
	0.02±0.06	0.22±0.11* (0.01)	0.05±0.06	4.95	7.4	1.9	0.03±0.03	0.16±0.05
	0.03±0.06	0.22±0.11* (0.01)		4.20	2.4	0.6	0.03±0.03	0.16±0.05
	0.02±0.06		0.05±0.06	0.93	5.7	3.8	0.03±0.03	0.17±0.05
		0.22±0.11* (0.01)	0.05±0.05	4.80	1.8	0	0.03±0.03	0.16±0.05
	0.03±0.05			0.27	0.6	2.5	0.03±0.03	0.17±0.05
			0.05±0.05	0.83	0	1.9	0.03±0.03	0.17±0.05

Cells under Fixed factors show parameter estimates ± SE. Values in parentheses are estimated effect sizes (Cohen’s f^2^). For target ethnicity, Non-White = 0, White = 1. Cells under Random factors show estimated variance ± SE.

* p < 0.05.

^a^Bayes Information Criterion difference from best model.

^b^Aikaike Information Criterion difference from best model.

^c^Not applicable: no model significantly predicts outcome variable.

Across all judges, in the video with full audio condition, M ± SD absolute difference between LSRP and LORP was 0.98 (± 0.76) for primary psychopathy and 1.08 (± 0.77) for secondary psychopathy (multi-level mixed model, ß ± SE = 0.09 ± 0.06, p = 0.09). In the silent video condition, the LSRP-LORP difference was 1.14 (± 0.88) for primary psychopathy and 1.16 (± 0.87) for secondary psychopathy (ß ± SE = -0.08 ± 0.06, p = 0.18). In the low pass filtered condition, LSRP-LORP difference was 1.11 (± 0.85) for primary psychopathy and 1.04 (± 0.78) for secondary psychopathy (ß ± SE = -0.07 ± 0.06, p = 0.27).

We also tested models in which the predictor variables were judge general trust, target LSRP score, and their interaction. In the video with full audio condition, for both primary psychopathy (ß = 0.146 ± 0.036, p < 0.0001, Cohen’s f^2^ = 0.07) and total psychopathy (ß = 0.148 ± 0.036, p < 0.0001, f^2^ = 0.07), the interaction term was significantly related to accuracy. Higher trusting judges made less accurate judgments with respect to targets higher in self-reported psychopathy. For secondary psychopathy, we found no relationship between the interaction term and accuracy (ß = 0.064 ± 0.040, p = 0.10). We found no relationships between the interaction term and accuracy in the silent video or the low passed filtered video conditions.

### Models predicting standard difference scores in psychopathy judgments

Tables 10–12 show models in which judge general trust and EQ scores are predictors of the standard difference score (SDS), i.e. the difference between LORP *z* score and LSRP *z* score. Both general trust and EQ were significantly negatively related to primary psychopathy SDS in the silent video condition (Table 10), i.e. relative to targets’ self-reports, judges higher in these traits judged targets to be lower in psychopathy than did judges lower in these traits. With respect to secondary psychopathy (Table 11), judge general trust was negatively related to SDS in the video with full audio condition, whereas judge EQ was negatively related to SDS in the silent video condition. With respect to total psychopathy (Table 12), judge general trust was negatively related to SDS in both the video with full audio and the silent video conditions, whereas judge EQ was negatively related to SDS in the silent video condition.

**Table 10:** Multilevel mixed regression models predicting LORP-LSRP standard difference score for primary psychopathy.

	Fixed factors					Random factors	
Condition	Judge trust	Judge EQ	Model χ^2^	ΔBIC^a^	ΔAIC^b^	Judge ID	Target ID
**Video with Full Audio**							
	-0.15±0.09	-0.08±0.11	3.95	N.A.^c^	N.A.^c^	0.21±0.07	0.87±0.15
	-0.16±0.09		3.32	N.A.^c^	N.A.^c^	0.21±0.07	0.87±0.15
		-0.11±0.11	1.01	N.A.^c^	N.A.^c^	0.23±0.07	0.87±0.15
**Silent Video**							
	-0.31±0.07*** (0.06)	-0.15±0.06** (0.02)	24.30***	0	0	0.06±0.04	1.17±0.20
	-0.31±0.08***		14.48***	0.2	4.0	0.09±0.05	1.15±0.20
		-0.14±0.07*	4.02*	8.5	12.3	0.15±0.06	1.16±0.20
**Low-pass Filtered Video**							
	0.04±0.08	-0.03±0.09	0.26	N.A.^c^	N.A.^c^	0.08±0.06	1.02±0.18
	0.03±0.08		0.16	N.A.^c^	N.A.^c^	0.08±0.06	1.02±0.18
		-0.01±0.08	0.03	N.A.^c^	N.A.^c^	0.08±0.06	0.99±0.18

Cells under Fixed factors show parameter estimates ± SE. Values in parentheses are estimated effect sizes (Cohen’s f^2^). Cells under Random factors show estimated variance ± SE.

* p < 0.05.

** p < 0.01.

*** p < 0.001.

^a^Bayes Information Criterion difference from best model.

^b^Aikaike Information Criterion difference from best model.

^c^Not applicable: no model significantly predicts outcome variable.

**Table 11:** Multilevel mixed regression models predicting LORP-LSRP standard difference score for secondary psychopathy.

	Fixed factors					Random factors	
Condition	Judge trust	Judge EQ	Model χ^2^	ΔBIC^a^	ΔAIC^b^	Judge ID	Target ID
**Video with Full Audio**							
	-0.16±0.07* (0.01)	-0.07±0.08	6.30*	5.0	1.3	0.07±0.04	0.96±0.17
	-0.17±0.07*		5.45*	0	0	0.07±0.04	0.96±0.28
		-0.10±0.09	1.28	3.9	3.9	0.09±0.05	0.97±0.27
**Silent Video**							
	0.09±0.08	-0.14±0.06* (0.01)	6.90*	4.4	0.6	0.05±0.04	0.92±0.17
	0.09±0.08		1.09	3.6	3.6	0.08±0.05	0.90±0.17
		-0.14±0.06*	5.18*	0	0	0.06±0.05	0.91±0.17
**Low-pass Filtered Video**							
	0.06±0.09	0.07±0.09	1.69	N.A.^c^	N.A.^c^	0.11±0.06	0.86±0.16
	0.08±0.08		1.01	N.A.^c^	N.A.^c^	0.11±0.06	0.86±0.16
		0.09±0.09	1.14	N.A.^c^	N.A.^c^	0.11±0.06	0.85±0.15

Cells under Fixed factors show parameter estimates ± SE. Values in parentheses are estimated effect sizes (Cohen’s f^2^). Cells under Random factors show estimated variance ± SE.

* p < 0.05.

^a^Bayes Information Criterion difference from best model.

^b^Aikaike Information Criterion difference from best model.

^c^Not applicable: no model significantly predicts outcome variable.

**Table 12:** Multilevel mixed regression models predicting LORP-LSRP standard difference score for total psychopathy.

	Fixed factors					Random factors	
Condition	Judge trust	Judge EQ	Model χ^2^	ΔBIC^a^	ΔAIC^b^	Judge ID	Target ID
**Video with Full Audio**							
	-0.17±0.08* (0.01)	-0.08±0.10	5.73	5.1	1.2	0.15±0.06	0.93±0.16
	-0.18±0.08*		4.87	0	0	0.15±0.06	0.92±0.16
		-0.12±0.10	1.31	3.3	3.3	0.17±0.06	0.93±0.16
**Silent Video**							
	-0.25±0.07*** (0.04)	-0.16±0.06** (0.03)	18.8***	0	0	0.05±0.04	1.10±0.19
	-0.25±0.08***		8.99**	1.0	4.7	0.09±0.05	1.09±0.20
		-0.16±0.07*	5.29*	4.1	7.8	0.11±0.06	1.10±0.19
**Low-pass Filtered Video**							
	0.06±0.09	0.01±0.09	0.54	N.A.^c^	N.A.^c^	0.10±0.06	0.94±0.17
	0.06±0.08		0.52	N.A.^c^	N.A.^c^	0.10±0.06	0.94±0.17
		0.03±0.08	0.11	N.A.^c^	N.A.^c^	0.10±0.06	0.94±0.17

Cells under Fixed factors show parameter estimates ± SE. Values in parentheses are estimated effect sizes (Cohen’s f^2^). Cells under Random factors show estimated variance ± SE.

* *p* < .05.

^a^Bayes Information Criterion difference from best model.

^b^Aikaike Information Criterion difference from best model.

^c^Not applicable: no model significantly predicts outcome variable.

## Discussion

The present study is the first to examine directly whether the self-reported psychopathy levels of non-institutionalized target individuals can be detected based solely on behavioral thin slices. We predicted that (1) judges would be more accurate assessing primary psychopathy than secondary psychopathy, (2) judges higher in general trust would rate targets as lower in psychopathic traits, (3) judges higher in general trust would make less accurate judgments, (4) judge trust and target psychopathy level would interact, such that more trusting judges would make less accurate judgments about targets higher in psychopathy, and (5) more empathetic judges would make more accurate judgments. Prediction (4) was supported with a small effect size. Support for the other predictions was mixed, and in no case very strong (Cohen’s f^2^ ≤ 0.02, i.e. negligible). We did find suggestive evidence for accuracy in judgments of primary psychopathic traits among undergraduates, based solely on video thin slices of non-directed zero-acquaintance social interaction. This finding is noteworthy because our video stimuli, depicting a low-stakes “small talk” experimental group discussion situation, were probably less informative about targets’ “dark” dispositions than the NASA Game task used by Rauthmann [31], which specifically elicited cooperative or competitive behavior.

Our judges’ ratings of targets’ primary psychopathy levels were significantly influenced by self-reported psychopathy only when judges had access to targets’ verbal as well as visible behavior. In contrast, Fowler et al. [30] found greater judgment accuracy of convicted criminals’ psychopathy levels for silent video than for video accompanied by sound. It may be that visible cues of psychopathy are more informative at the higher levels of psychopathy found in a prison population than in an undergraduate population. It may also be that audio cues are less informative in thinner slices (5–10 second vs. our 1.5 minute) or in monologues (compared with our conversations).

We predicted that judgments of primary psychopathy would be more accurate than judgments of secondary psychopathy. We found only a trend in this direction in the video with full audio condition, and trends in the opposite direction in the silent and low pass filtered condition. Possibly, the impulsivity characteristic of secondary psychopathy manifests itself during zero-acquaintance situations in extra-semantic speech patterns such as a louder speaking voice. In the absence of access to the semantic content of targets’ speech, judges might focus on this feature. Elsewhere [44] we report that in an unannounced, post-conversation Prisoner’s Dilemma game played for small monetary rewards, targets’ levels of secondary psychopathy, but not primary psychopathy, was associated with a higher probability of receiving cooperation from co-participants. Possibly, the same non-verbal or extra-semantic behavioral features that promote recognition of secondary psychopathy by judges also elicit greater cooperation from interlocutors. Judgments of total psychopathy (i.e. the entire LSRP/LORP scale) were not significantly accurate in any experimental condition.

We predicted that judges who were both high in General Trust and low in Caution would judge targets as lower in psychopathy. The low internal reliability of the Caution scale precluded testing this prediction. This result is probably not attributable to a statistical fluke or to peculiar characteristics of our participants, as a recent study [50] reported a Cronbach’s alpha of 0.32 for this scale in a Canadian student sample. We examined main effects of judge General Trust and found that it independently negatively predicted perceived (1) primary and total psychopathy levels in both the video with full audio and silent video conditions and (2) secondary psychopathy in the video with full audio condition. However, in the video with full audio condition, the best fitting models predicting judgments of primary psychopathy either did not include general trust (BIC), or did include general trust but not as a significant effect.

Our most surprising finding was the consistent relationship, across all three stimulus conditions, between target ethnicity and psychopathy judgments, such that White and biracial White-Non-White target individuals were perceived as higher in psychopathic traits than non-White targets. Self-reported psychopathy did not differ significantly between the two groups, although there were weak trends in the same direction as the video-based judgments, and this accounts for our general finding that judgment accuracy was not related to target ethnicity. Interpretation of these results is complicated by our lack of data on judges’ ethnic identity. Ethnic and national personality stereotypes are widespread and mostly inaccurate [51, 52]. Additional research is necessary to replicate our findings regarding ethnicity and perceived psychopathy, and to test hypotheses about the cause(s) of the observed patterns.

Considering the effects of individual dispositions on person perception [17, 18, 19], it is unsurprising that judges’ views of the overall prevalence of benign motives in humans would affect their inferences about the psychopathy levels of people about whom they have very limited information. Using an accuracy criterion of absolute value of the difference between judge’s rating and self-rating (Tables 7–9), in our video with full audio condition, our findings provided tepid support for our prediction of a main effect of judge general trust on accuracy in judging primary psychopathy. We did not predict, before beginning analysis, that the interaction between judge general trust and target self-reported psychopathy would affect judgment accuracy. However, this prediction is consistent with our general approach, as further informed by error management theory [22, 36]. Overall, judges’ ratings of targets’ psychopathy levels were much higher than targets’ self-reports. Using standard difference scores (i.e. self-report rating subtracted from judge’s rating) [47] as a dependent measure, more trusting judges showed smaller positive (or larger negative) differences between their ratings and self-report ratings for primary psychopathy in the silent video condition, secondary psychopathy in the video with full audio condition, and total psychopathy in both conditions. In the video with full audio condition, higher trusting judges made less accurate judgments with respect to targets higher in self-reported primary and total psychopathy, with small effect sizes. We interpret these results as suggesting that people low in general trust overestimate the psychopathy levels of most people, but that people high in general trust underestimate the psychopathy levels of those few people who are in high in psychopathic traits (and therefore most prone to exploit social partners).

One hypothesis for the persistence of primary psychopathic traits is that they represent an evolved exploitative strategy that can thrive when rare because the costs of detecting psychopathic traits in others are not worth paying when these traits are rare ([21]; see also [53]). In other words, psychopathy persists as a result of negative frequency-dependent selection. We have presented evidence for a modified model of subclinical primary psychopathy, that it represents a social strategy of selective cooperation with high-value partners rather than an obligately exploitative strategy [44]. In accord with this model, evidence from a study of personality and behavior in a trust game suggests that, in the absence of information about a prospective social partner’s value, psychopathic traits are unrelated to investment in a partner (i.e. to the first player’s decision), whereas reciprocation of the investment (i.e. the second player’s decision to return a portion of the money) is less likely from more psychopathic individuals [54]. An implication of both models, however, is that natural selection should generate an “arms race” between the ability to detect psychopathic traits in others and the ability to hide them from others. We suggest that the “glib charm” or “mask” [1] of those high in psychopathic traits is intermittently penetrable, and possibly only by certain observers. The results of the present study are generally consistent with this scenario: primary psychopathic traits were detectable at slightly better than chance levels, and only when judges had access to targets’ verbal as well as visual behavior. Furthermore, our finding of individual differences in the ability to detect primary psychopathy is also consistent with, though not a test of, the operation of negative frequency-dependent selection on psychopathy operating through the costs of psychopathy-detection. More trusting people are worse than less trusting people at detecting psychopathy in others, in the sense that their judgments deviate more from the self-described psychopathy levels of people they encounter. However, because more trusting individuals are reluctant to attribute callousness, dishonesty or manipulativeness to others based on limited information, they can presumably better reap the benefits of mutually beneficial cooperation in some social ecologies without paying the costs of psychopathy detection (see [55]). A similar argument has been made [56] for Big Five agreeableness, which includes trust as one of its facets [57]. An important caveat, however, is that general trust may be more adaptively malleable than is assumed by models that attribute variation in this trait to a mix of genetic variation and irreversible early environmental input; even brief exposure to cues of pervasive low social investment reduces adults’ trust levels outside of personal relationships [58].

Surprisingly, more empathetic judges did not make more accurate judgments of targets’ psychopathy levels when accuracy was measured as the absolute value of the difference between judge’s rating and self-rating. Indeed, in the silent video condition their performance was actually worse than that of less empathetic judges. When accuracy was measured as SDS, more empathetic judges, like more trusting judges, showed smaller positive (or larger negative) discrepancies between their ratings and self-report ratings for primary and total psychopathy in the silent video condition. Empathy is generally associated with greater interpersonal sensitivity [38]. However, empathy as measured by the EQ encompasses cognitive and affective responses to others’ inferred momentary emotional states [39], which are distinct from others’ enduring personality traits. Because shallow emotions comprise one aspect of psychopathy [1, 2], it is possible that individuals high in psychopathic traits do not provide enough behavioral material to give highly empathetic individuals an advantage in judging their psychopathy levels based on thin slices. As tentative support for this speculation, among our target individuals, analysis using the Linguistic Inquiry and Word Count application [59] showed that F1 LSRP was negatively associated with the use of affect words, after controlling for sex [34]. Perhaps the negative effect of EQ on judgment accuracy in the silent video condition occurred because of the condition’s induced focus on non-verbal emotional cues. The EQ taps the ability to read such cues [60]. With respect to judgments of psychopathy, as distinct from other judgments, more empathetic judges might be particularly prone to inaccuracy when provided with only non-verbal cues.

This study had a number of limitations. First, our measure of judgment accuracy was self-other agreement, a less stringent criterion than agreement between lay other-ratings and clinical assessment (as was used in [30]). Second, our theoretical framework relied on the distinction between psychopathy’s underlying propensity toward exploitative behavior and its superficial “mask of sanity” [1], but the LSRP-LORP, unlike the Psychopathic Personality Inventory [61], does not distinguish between these sub-traits. However, target individuals higher in LSRP F1 tended to dominate triadic conversations [34], suggesting that LSRP F1 captures some of the same variation captured by the PPI’s Fearless Dominance scale, which is hypothesized to represent the “mask” ([62] but see [63]). Third, we did not collect information on judges’ ages or ethnicities, limiting our ability to test adaptive error management hypotheses of psychopathy detection, particularly in view of our findings regarding the effects of targets’ ethnicity. For example, we cannot test whether judges erred more toward false alarms than toward misses when describing targets of different ethnicities from their own, compared to when judging targets of their own ethnicity. Fourth, for all judges, the self-report instruments were administered before they viewed the video of the targets. An alternative, counter-balanced, design would have addressed the possibility that answering questions about trust, caution and empathy generates bias in subsequent judgments about others’ psychopathic traits. Fifth, our regression models included different versions (self- vs. other-report) of the same instrument on both sides of regression equations in Tables 4–6, potentially inflating parameter estimates. Sixth, all our judges’ ratings were made based on a single slice length of 1.5 minutes. Fowler et al. [30] actually found greater accuracy in detecting psychopathy traits from 5 second and 10 second slices than from 20 second slices. Possibly, the judges higher in General Trust initially judged targets to be higher in psychopathy than our results indicate, before disregarding their own initial impressions and gradually lowering their estimates during the 1.5 minute slice. Future research could test this hypothesis using a tool such as the Continuous Affect Rating and Media Notation software package [64], with which judges can provide moment by moment ratings of video stimuli. Seventh, because the LORP was the only instrument by which our judges rated our targets, we cannot test the alternative hypothesis that General Trust is associated with heightened positivity with respect to judging all personality traits (not just psychopathy) for which one pole is more socially desirable than the other. Eighth, we did not address the question of which behaviors of targets were used by judges to reach their inferences about targets’ psychopathy levels. Judges were not merely presuming that targets pursuing academic majors associated with material acquisitiveness were higher in primary psychopathy. Previous analyses of the stimulus videos [34] showed that individuals higher in primary psychopathy dominated the interactions with respect to proportion of words uttered, interruptions, and topic control. However, none of the LSRP/LORP items is directly related to conversational dominance, so unless judges possessed a folk model linking conversational dominance to callous affect and interpersonal manipulation, variation in targets’ conversational dominance cannot have influenced LORP scores. Because judgments of primary psychopathy were significantly accurate only in the video with full audio condition, future research should focus on how the semantic content of quotidian speech (e.g. expressions of arrogance, contempt for others, and disrespect for laws and rules) varies as a function of subclinical primary psychopathy. Word category use frequency might also vary with psychopathy [34, 65], as has been shown for another Dark Triad trait, narcissism [66].
